# Macroporous PEG-Alginate Hybrid Double-Network Cryogels with Tunable Degradation Rates Prepared via Radical-Free Cross-Linking for Cartilage Tissue Engineering

**DOI:** 10.1021/acsabm.4c00091

**Published:** 2024-08-13

**Authors:** Kaixiang Zhang, Zining Yang, Michael Patrick Seitz, Era Jain

**Affiliations:** † Department of Biomedical and Chemical engineering, Syracuse University, Syracuse, New York 13244, United States; ‡ Bioinspired Syracuse: Institute for Material and Living System, Syracuse University, Syracuse, New York 13244, United States

**Keywords:** cryogels, double network, cartilage tissue engineering, hydrogels, click reaction

## Abstract

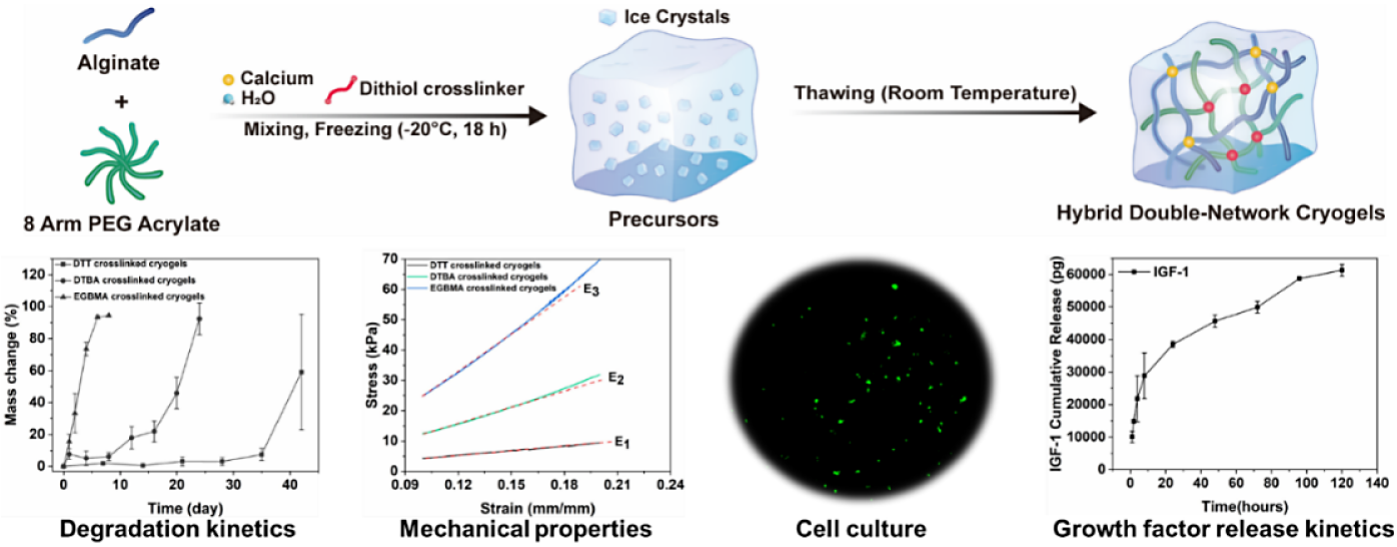

Trauma or repeated damage to joints can result in focal cartilage defects, significantly elevating the risk of osteoarthritis. Damaged cartilage has an inherently limited self-healing capacity and remains an urgent unmet clinical need. Consequently, there is growing interest in biodegradable hydrogels as potential scaffolds for the repair or reconstruction of cartilage defects. Here, we developed a biodegradable and macroporous hybrid double-network (DN) cryogel by combining two independently cross-linked networks of multiarm polyethylene glycol (PEG) acrylate and alginate.Hybrid DN cryogels are formed using highly biocompatible click reactions for the PEG network and ionic bonding for the alginate network. By judicious selection of various structurally similar cross-linkers to form the PEG network, we can generate hybrid DN cryogels with customizable degradation kinetics. The resulting PEG-alginate hybrid DN cryogels have an interconnected macroporous structure, high mechanical strength, and rapid swelling kinetics. The interconnected macropores in the cryogels support efficient mesenchymal stem cell infiltration at a high density. Finally, we demonstrate that PEG-alginate hybrid DN cryogels allow sustained release of chondrogenic growth factors and support chondrogenic differentiation of mouse mesenchymal stem cells. This study provides a novel method to generate macroporous hybrid DN cryogels with customizable degradation rates and a potential scaffold for cartilage tissue engineering.

## 1 Introduction

Trauma or repeated injury to the joint can result in focal cartilage defects, which significantly increase the risk of osteoarthritis, a painful and disabling disease of the joint. Early-stage treatment of articular cartilage defects can be an effective strategy for reducing the incidence of arthritis. However, damaged articular cartilage has inherently limited self-healing capacity and remains an urgent unmet clinical need.^1,2^ Clinically used therapies for articular cartilage repair involve autologous chondrocyte (ACI) implantation, microfracture, and allogeneic osteochondral transplantation. However, these therapeutic approaches have achieved very limited success due to the generation of fibrocartilage within the defect site, high risk of donor site morbidity, and requirement for multiple surgeries.^3−5^

Hydrogels have become a popular choice as potential scaffolds to replace damaged cartilage or as cell carriers to promote cartilage repair.^6^ Recently, there has been a growing interest in the use of hybrid double-network (DN) hydrogels for cartilage tissue engineering due to their high toughness compared to conventional hydrogels.^7−9^ Unlike conventional DN hydrogels, hybrid DN hydrogels are made with one covalently cross-linked synthetic polymer and a second ionically cross-linked natural polymer. Replacement of one of the covalently cross-linked networks with an ionically cross-linked network leads to high toughness, stretchability, and self-healing capabilities.^7−11^

Despite the high toughness, the nanoporous and nondegradable nature of the current DN hydrogels limits their applications in cartilage tissue engineering. The lack of interconnected macroporous structures and biodegradation in most DN hydrogels leads to poor cell and tissue infiltration.^12^ Several studies have shown that macroporous structure and degradability are important for cell infiltration and extracellular matrix (ECM) deposition in cartilage constructs.^13−17^

An emerging, simplistic, and biocompatible method to fabricate hydrogels with a macroporous structure is “cryogelation.” Cryogels are gel matrices with interconnected macroporous structures synthesized by “cryogelation,” which is the cross-linking of gel precursors at subzero temperatures.^18^ Under subzero temperature, most of the solvent freezes, while a small part of the solvent remains nonfrozen (NFLP), where most of the gel precursor remains cryoconcentrated in a small volume, and the polymerization or gelation proceeds at an accelerated pace around the ice crystals.^19^ The formation of interconnected pores is due to the formation and melting of nucleated ice crystals in the water phase during the freezing and thawing processes, respectively. Due to their interconnected porous structure, cryogels have high elasticity, quick swelling kinetics, and very high water uptake.^20,21^ Compared to traditional hydrogels, the macroporous structures of cryogels allow unimpeded convective mass transfer of solutes, including efficient cell infiltration, and long-term cell culture at a very high density.^22−24^ Moreover, due to their unique structure and properties, cryogels can withstand high compression loads and can undergo cyclic mechanical loading without loss of structural integrity.^25,26^ Thus, they are presented as potential scaffolds for cartilage tissue engineering.

Alginate is a naturally occurring anionic polymer that is biocompatible, has low toxicity, is of relatively low cost, and can be gelled by adding divalent cations such as calcium.^27^ Alginate hydrogels have been commonly used for chondrocyte culture and cartilage tissue engineering owing to their structural similarity to sulfated glycosaminoglycans, a critical component of the ECM constituting cartilage.^28^ Although alginate hydrogels are commonly made using ionic gelation between alginate and divalent metal ions, alginate cryogels have been produced only using free-radical polymerization of methacrylated alginate or via carbodiimide chemistry,^29−31^ rendering these macroporous networks nonbiodegradable. Fabrication of alginate networks via ionic gelation under subzero conditions allows the formation of a biodegradable macroporous cryogel network while using a nontoxic and biocompatible aqueous-based cross-linking reaction. However, ionically cross-linked alginate alone leads to the formation of mechanically weak gels.^30,31^ Thus, combining alginate with another synthetic polymer to form a macroporous hybrid double network reinforces the mechanical properties of the resulting cryogel.

Polyethylene glycol (PEG)–based hydrogels, due to their high biocompatibility and inertness, are popularly used in tissue engineering and drug delivery applications.^32,33^ Despite their advantages and common use as hydrogels, only a few PEG-based macroporous scaffolds, particularly cryogels, have been reported in the literature. Most PEG cryogels reported are made using free-radical polymerization of acrylate-terminated PEG chains, resulting in a nondegradable cryogel network.^34−36^ PEG hydrogels made via click reaction (Michael-type addition) between thiol and acrylate (an electron-poor ene)–terminated multiarmed PEG polymers result in the formation of thioether-ester links at each cross-link, which are hydrolytically degradable. The reaction involves the formation of a thiolate ion in the presence of a base, which then reacts with the electron-poor “ene” that is acrylate with high specificity. The reaction proceeds with high efficiency in aqueous buffer under physiologically relevant conditions, thus making it highly biocompatible.^37−40^ The rate of degradation in these hydrogels can be controlled by the chemical identity of the thiol cross-linker. Regulating the hydrogel degradation through minor modifications in the cross-linker structure is an effective strategy to obtain scaffolds of desired degradation rate for tissue engineering applications.^38,41,42^

**Figure 1:**
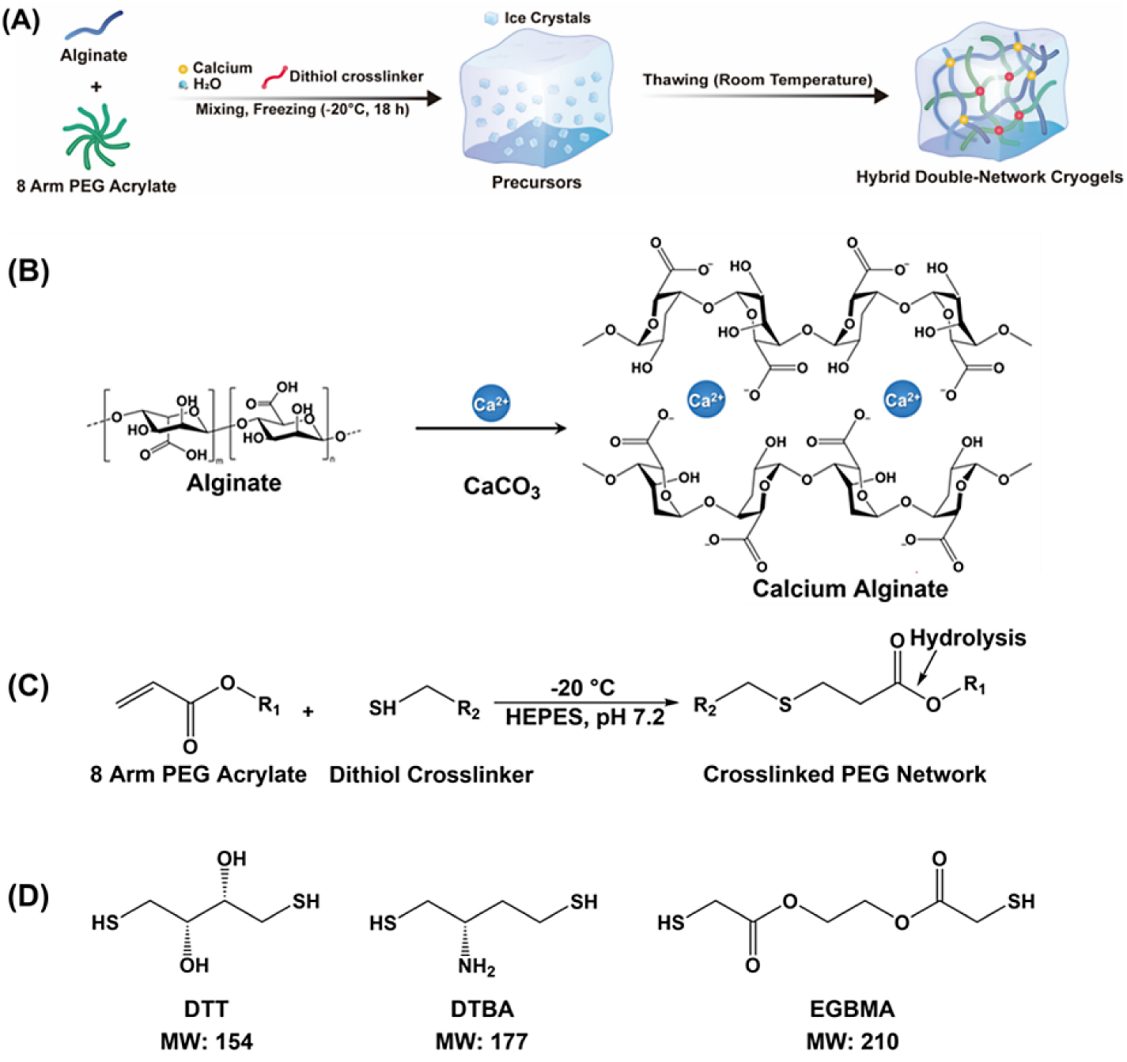
(A) Schematic of the synthesis procedure for the PEG-alginate hybrid double-network cryogel: alginate and cross-linker calcium carbonate, 8-arm PEGAc, and dithiol cross-linker are dissolved in a buffer, mixed, and incubated at subzero temperature. Ice crystals are formed in the precursor solution upon freezing. After the gelation is completed, the mixture is thawed at room temperature. The ice crystals melt away, leaving behind an interconnected macroporous network. (B) Chemical reaction indicating ionic gelation for the formation of alginate gels. A double displacement reaction occurs in which calcium ions replace sodium ions on alginate, producing calcium alginate. (C) Chemical reaction scheme for the formation of the PEG network. Michael-type additions between deprotonated forms of dithiol cross-linkers (EGBMA, DTT, and DTBA) and terminal acrylate bonds (e.g., 8-arm PEGAc) form hydrolytically degradable cross-link. The reaction involves the formation of a thiolate ion in the presence of a base, which then reacts with an unsaturated acrylate with high specificity. (D) Chemical structure and molecular weight of dithiol cross-linkers used for the synthesis of the PEG network in hybrid DN cryogels.

In this study, we synthesized macroporous and biodegradable hybrid DN cryogels of PEG and alginate using highly biocompatible click chemistry for the formation of covalent networks and ionic bonding for the formation of noncovalent networks (Figure 1). We were the first to synthesize PEG-alginate hybrid DN cryogels by combining a click reaction (Michael addition) and ionic gelation. The resulting hybrid DN cryogels have an interconnected network of macropores, high mechanical strength, and fast swelling kinetics. Moreover, using a variety of structurally similar cross-linkers (Figure 1) to form the PEG network, we were able to generate hybrid DN cryogels with customizable degradation rates. Furthermore, the hybrid DN cryogels exhibited sustained release of potent growth factors as well as support culture and chondrogenic differentiation of mouse mesenchymal stem cells. These characteristics make PEG-alginate hybrid DN cryogels potential scaffolds for cartilage tissue engineering.

## 2 Materials and Methods

### 2.1 Materials

8-Arm-PEG acrylate (8-arm PEGAc,10 kDa, Jenkem Technology, USA Plano, TX), ethylene glycol bis(mercaptoacetate) (EGBMA) and (S)-2-aminobutane-1,4-dithiol (DTBA, Sigma-Aldrich, Saint Louis, MO), sodium alginate (M/G ratio 0.6; GMB, Manugel, FMC Biopolymer, Philadelphia, PA), calcium carbonate (CaCO_3_, 99+%) and glucono-delta-lactone (GDL, 99%, Acros Organics, NJ, USA), *N*-2-hydroxyethylpiperazine-*N*-2-ethanesulfonic acid (HEPES), fetal bovine serum (FBS), penicillin streptomycin (PS), l-glutamine, Dulbecco’s phosphate-buffered saline (DPBS), Dulbecco’s modified Eagle medium (DMEM/F12 (1:1)), trypsin-EDTA (0.05%), collagen I (rat tail), acetic acid, dithiothreitol (DTT), transforming growth factor-beta 1 (TGF-β1), and LIVE/DEAD viability/cytotoxicity kit (Thermo Fisher Scientific), insulin-like growth factor 1 (IGF-1, Peprotech, Cranbury, NJ), ascorbic acid (Thermo Fisher Scientific), dexamethasone (Gibco, Grand Island, NY), insulin-transferrin-selenium (ITS (1X), Sigma-Aldrich, Saint Louis, MO), and bone morphogenetic protein 2 (BMP-2, Peprotech, Cranbury, NJ).

### 2.2 Synthesis of PEG-Alginate Hybrid Double-Network Cryogels

Hybrid DN cryogels were synthesized through simultaneous cross-linking of two polymeric networks composed of alginate and 8-arm PEGAc. First, alginate was dissolved in 100 mM HEPES buffer (pH 7.4) at room temperature (RT) for at least 24 h to obtain a 1.25% w/v alginate solution. 8-arm PEGAc was added to the alginate to obtain a 20% w/v solution. The 8-arm PEGAc-alginate solution was vortexed for 20 s and then centrifuged at 3000 rpm for 5 min to remove any entrapped bubbles. A 10× cross-linker stock solution was prepared by mixing 0.3 M CaCO_3_, 0.6 M GDL, and 0.8 M of one of the three dithiol cross-linkers (DTT, DTBA, and EGBMA; Figure 1D) in HEPES buffer. All solutions were cooled to 4 °C and maintained on ice until used. The 10× cross-linker solution was added to the 8-arm PEGAc-alginate solution. The mixture was quickly vortexed for 15 s and placed immediately in a cryostat bath. To cross-link the 8-arm PEG Ac network, any of the three dithiol cross-linkers (DTT, DTBA, and EGBMA; Figure 1C) was added at a molar ratio of 1:1 acrylate and thiol. The final concentration of alginate was 1% (w/v), and 8-arm PEGAc was 20% (w/v). The gelation of all the three cryogel precursors was carried out in a thermostatic bath maintained at −20 °C for at least 18h. The formed cryogels were thawed and washed at RT in deionized water for 15 min, air dried, and stored in 20% w/v of ethanol for further experiments.

### 2.3 Scanning Electron Microscopy

For scanning electron microscopy (SEM) analysis, all DN hybrid cryogels were dried in a methanol gradient. Briefly, the samples were cut into 5 mm-high discs and dehydrated by placing them in an increasing gradient of ethanol (20%, 40%, 60%, 80% v/v) for 5 min each. Finally, the samples were placed in 100% (v/v) ethanol for 30 min for complete dehydration. The samples were then vacuum dried overnight using a lyophilizer before gold plating. Dried and gold-coated cryogels were imaged using a JEOL JSM 5600 SEM. Cross-sections of cryogels from the top, middle, and bottom parts were imaged. Pore sizes were quantified using threshold values and measured particle functions in ImageJ.

### 2.4 Swelling Kinetics of Cryogels

The swelling kinetics of all cryogels were measured using a conventional gravimetric method. Briefly, each cryogel sample (5 mm in length and 6 mm in diameter) was dried in an alcohol gradient. Dried cryogel samples were placed in phosphate-buffered saline (PBS) solution (with 2 mM CaCl_2_) and maintained at 37 °C with gentle rocking. The water absorption rate was measured by weighing the samples at regular time intervals and by calculating the changes in mass over time. Before each measurement, the cryogels were removed from the swelling medium, and excess water on the surface was removed by Kimwipe. The cryogels were weighed at 2 min, 5 min, 30 min, 1 h, and 24 h and then every 48 h until equilibrium was reached. All experiments were conducted in triplicate. The swelling ratio of the cryogels was determined using the equation:
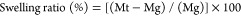


where Mt is the swollen mass of cryogels at a given time interval and Mg is the mass of the air-dried cryogels.

All samples were tested in triplicate. The swelling rate was calculated by performing a linear regression of the swelling ratio/time data using the quick fit function in Origin.

### 2.5 Degradation Kinetics of Cryogels

All cryogels were dehydrated in an increasing gradient of methanol (20%, 40%, 60%, 80%, and 100% v/v). On day 0, the initial dry mass of the cryogels was recorded (*W*_0_). After sterilization, the cryogels were incubated in complete culture media consisting of DMEM-F12 supplemented with 10% FBS and 1% penicillin–streptomycin at 37 °C with 5% CO_2_. Excess water on the cryogel surface was wiped with KimWipe, and the swollen mass of cryogels (*W*_s_) after 2 h was measured for all cryogels. Furthermore, at regular time intervals for each type of cryogel, swollen mass (*W*_s_) and dry mass (*W*_d_) were obtained. Three replicates were measured at each time point. The degradation degree was evaluated by the mass change using the following equation: Mass change (%) = (*W*_0_–*W*_d_)/*W*_0_ × 100%. All samples were tested in triplicate. The degradation rate was calculated by performing a linear regression on the mass change/time curve using the quick fit function in Origin.

### 2.6 Mechanical Analysis of Cryogels

A Shimadzu EZ-LX (346–57300–42) compression tester was used to perform the compression test. All samples were swollen to equilibrium in PBS before compression analysis. Cryogel samples with a diameter of 8 mm and length of 6 mm were placed between the two flat plates of the load frame. A preload of 0.01 N was applied to confirm the clear contact between the compressed plate and the cryogels. The samples were compressed to 70% of their total length at a speed of 1 mm/min using a 100 N load cell. The compression force was recorded, and the column length change caused by compression was measured. The following equations were used to estimate the compression modulus of the cryogels:
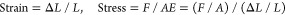


where *E* is the Young’s modulus of elasticity, *F* is the applied force, *A* is the cross-sectional area of the sample, *L* is the initial length of the sample, and Δ*L* is the change in length under compressive forces. The linear region of the stress versus strain graphs was analyzed to obtain the elastic modulus for each sample. Three samples per cryogel were analyzed, and the elastic modulus values were expressed as average ± standard deviation.

### 2.7 Rheological Measurements of Cryogels

The rheology test was conducted on TA-DHR3 rotational rheometer (TA Instruments, New Castle, USA) using 8-mm parallel plates. Cryogel samples (8 mm in diameter and a height of ∼6 mm) were incubated in 1 × PBS (with 2 mM CaCl_2_, pH 7.4) buffer until equilibrium was reached or for 2 h at 37 °C. Before the measurements, excess water from the cryogel surface was wiped off by KimWipe. Strain amplitude tests were conducted at a constant frequency of 1 rad/s over a strain range of 0.01–1%. Frequency sweep tests were set at a constant strain of 0.1% and in a frequency range of 0.1–100 rad/s. Three samples per cryogel were analyzed, and the storage and loss modulus values were expressed as the average ± standard deviation.

### 2.8 Growth Factor Loading and Release Kinetics Measurement

TGF-β1 30 μL (3.33 ng/mL) and IGF-1 30 μL (3.33 ng/mL) solutions prepared in DMEM-F12 were loaded into dehydrated cryogels (diameter of 4 mm and height ∼1.5 mm) in separate tubes. After overnight incubation, samples were washed and placed in 200 μL of DMEM-F12 media (with 1% penicillin streptomycin and 0.4% fungizone) on a shaker at 37 °C. All media in the tubes were collected at regular intervals and replaced with an equal volume of fresh media. The amount of TGF-β1 and IGF-1 released at each interval was quantified using standard ELISA according to manufacturer’s instructions. We utilized the modified Fick’s law to calculate the effective diffusion coefficient, *D*_e_, for short release times in slab geometry, as previously reported.^40^

### 2.9 Cell Maintenance, Collagen Coating, and Cell Culture in Cryogels

Clonally derived mouse mesenchymal stem cells (D1 mesenchymal stem cell American Type Cell Culture (ATCC); passages 8–15) were maintained in DMEM-F12 media supplemented with 10% FBS and 1% penicillin–streptomycin in a humidified 5% CO_2_ incubator at 37 °C. Upon reaching 80% confluence, cells were detached using 0.25% trypsin and 0.05% EDTA and centrifuged to obtain cell pellets. The harvested cells were resuspended in fresh DMEM-F12 medium and reseeded into a T-flask.

Cryogels (diameter of 4 mm and height of 1.5 mm) were sterilized with 70% ethanol and coated with collagen type I (50 μg/mL). The cryogels were then partially dehydrated under sterile conditions for 3 h. For cell seeding, 30 μL of cell suspension containing 1 × 10^5^ cells were seeded on the surface of each cryogel and incubated at 37 °C for 1 h to allow cell attachment. The cryogels were incubated in 200 μL of complete DMEM-F12 medium at 37 °C under 5% CO_2_. The media were refreshed every 48 h.

### 2.10 Cell Viability and Infiltration Measurement

To assess the biocompatibility of cryogels, the cell viability was determined using a live/dead assay. After D1 cells were cultured for 3 and 7 days, DMEM-F12 media were removed and the cell-loaded cryogel scaffold was washed three times with PBS solution to remove any unattached cells. The samples were then incubated with calcein-AM (green) and ethidium homodimer (red) for 45 min, according to the manufacturer’s protocol. Following washing of the excess dye, fluorescence images were captured using a Thunder upright microscope (Leica, Wetzlar, Germany) and analyzed using ImageJ software to calculate the ratio of the total viable cell area to the total viable and dead area. To measure cell infiltration, the cryogel scaffolds were fixed in 4% paraformaldehyde (PFA), stained with DAPI (in PBS 300 nM), and imaged using a Zeiss LSM 710 confocal microscope. The number of cells present throughout the cryogel samples was then quantified using ImageJ.

### 2.11 MSC Seeding in Cryogels and Chondrogenic Differentiation

D1 MSCs (passages 12–15) were maintained as described earlier. Cells were detached using trypsin-EDTA, centrifuged, and resuspended in fresh DMEM-F12 medium at the required seeding density. The cryogel samples were prepared as described earlier. A 30 μL of cell suspension containing 2 × 10^5^ cells was applied to the surface of each dehydrated cryogel and incubated at 37 °C for 1.5 h to allow cell attachment. The cryogels were then incubated in 300 μL of complete media at 37 °C with 5% CO_2_. The cryogels were divided into two groups: one group received DMEM-F12 supplemented with 10% FBS and the other group received chondrogenic medium (DMEM-F12 supplemented with 20 ng/mL TGF-β1, 10 nM dexamethasone, 50 nM ascorbic acid, and 1X ITS+1 premix (insulin, human transferrin, and selenous acid). Some additional samples also received chondrogenic media supplemented with bone morphogenetic protein–2 (BMP-2; 200 ng/mL). The media were refreshed every 48 h. Cells directly seeded in tissue culture plastic in DMEM-F12 with 10% FBS were used as controls. At each time point, three samples were removed and used for immunostaining and gene expression analysis.

### 2.12 RNA Extraction and Target Gene Expression

RNA (RNeasy total RNA kit, Qiagen, Valencia, CA) was extracted from the D1MSCs seeded in tissue culture plates and cryogels on D1, D7, and D14. RNA was then reverse transcribed to cDNA using an iSCRIPT cDNA synthesis kit (Bio-Rad, Hercules, CA). Quantitative polymerase chain reaction (qPCR) was then performed using 10 ng of cDNA and SYBER Green Master Mix in a QuantStudio 3 Thermocycler (Thermo Fisher). The primer sequences were used as reported in previous studies^43,44^ and were synthesized by Thermo Fisher Scientific. The primer sequences are listed in Table 1. The expression of collagen type 2 (COL2), aggrecan (ACAN), collagen type 1 (COL1), SRY-Box9 (SOX 9), and runt-related gene 2 (RUNX2) was determined and normalized to the housekeeping gene glyceraldehyde-3-phosphate dehydrogenase (GAPDH). Relative expression was calculated using the ΔΔCT method and expressed as fold change by normalizing to the expression levels of D1MSCs cultured in tissue culture plastic on day 1 (D1).

**Table 1:** Primer Sequences Used for Gene Expression Analysis

**specific primers**	**forward primer sequence**	**reverse primer sequence**
mCOL2	5′-CTGACCTGACCTGATGATACC-3′	5′-CACCAGATAGTTCCTGTCTCC-3′
mACAN	5′-CTCAGTGGCTTTCCTTCTGG-3′	5′-CTGCTCCCAGTCTCAACTCC-3′
mSox-9	5′-TACGACTGGACGCTGGTGCC-3′	5′-CCGTTCTTCACCGACTTCCTCC-3′
mCOL1	5′-TCAGAGGCGAAGGCAACAGTC-3′	5′-GCAGGCGGGAGGTCTTGG-3′
mRUNX2	5′-CCTGAACTCTGCACCAAGTC-3′	5′-GAGGTGGCAGTGTCATCATC-3′
mGAPDH	5′-TGAAGCAGGCATCTGAGGG-3′	5′-CGAAGGTGGAAGAGTGGGAG-3′

### 2.13 Immunostaining of Collagen Type 2

D1MSC-loaded cryogel scaffolds on days 1, 14, and 21 were removed from the media and washed three times with PBS. The samples were fixed in 4% PFA for 20 min, washed with PBS thrice, and blocked with 1% bovine serum albumin (BSA) containing 0.3% Triton X-100 for 60 min. The samples were washed three times with PBS and incubated with collagen II polyclonal antibody (PA1–26206; Thermo Fisher; 1:100) in PBS containing 1% BSA and 0.1% Tween overnight at 4 °C. Excess primary antibody was removed by washing with PBS three times, with each wash lasting 45 min. Samples were then incubated in a solution of secondary antibody Goat anti-Rabbit IgG Alexa Fluor 546 (1:500; Thermo Fisher) in PBS containing 1% BSA and 0.1% Tween and incubated overnight at 4 °C. After washing with PBS three times, with each wash lasting 45 min, each sample was counterstained with DAPI in PBS (300 nM) for 15 min. Finally, the samples were washed in PBS three times, with each was lasting10 min. Samples were imaged using a Zeiss LSM 710 confocal microscope. Immunostained images were analyzed using ImageJ.^45^ Briefly, each image was threshold to ensure consistency across all samples, and the mean fluorescence intensity (MFI) in the region of interest was determined by subtracting background fluorescence in unstained regions. The relative MFI was obtained by dividing the MFI of collagen 2 by that of DAPI. This ratio provides a normalized measure of collagen 2 expression relative to nuclear staining, allowing quantitative analysis.

### 2.14 Statistical Analysis

All the results were expressed as the average ± standard deviation from triplicate samples per experiment. One-way ANOVA followed by Tukey’s posthoc correction was used to compare more than two samples in a dataset unless indicated otherwise. A p value of less than 0.05 was considered statistically significant.

## 3 Results and Discussion

### 3.1 Synthesis of PEG-Alginate Hybrid DN Cryogels

In this study, we first optimized different synthesis parameters for the formation of the hybrid DN cryogel (Table S1). The first parameter we studied was the synthesis temperature to form hybrid DN cryogels. Temperature is a critical parameter for the synthesis of cryogels as it profoundly influences the gelation process, affecting the kinetics of polymerization and structural properties of the resulting cryogel, thereby playing a pivotal role in determining the material’s porosity, pore size, mechanical strength, and overall performance.^18,46^ We chose three different synthesis temperatures, −8 °C, −12 °C, and −20 °C, for the synthesis of cryogels. Qualitatively comparing the cryogels prepared at different temperatures, the cryogels prepared at −20 °C were opaque and elastic and had higher mechanical integrity than the cryogels prepared at higher temperatures. Thus, −20 °C was considered to be the optimal temperature for cryogel synthesis.

Next, we screened 4-arm and 8-arm PEG acrylates (10 kDa) for their ability to formrobust cryogel networks. Our preliminary data indicate that both 4-arm and 8-arm PEG acrylates can form a robust cryogel network, with those formed using 8-arm acrylates exhibiting relatively higher porosity and mechanical strength. We chose multiarm PEG acrylates as previous studies by our group and others have demonstrated that multiarm PEG acrylates form robust hydrogels under physiological conditions and enable the incorporation of cell-instructive peptides without significantly affecting the cross-linking efficiency. Moreover, varying the number of arms offers further control over the degradation rate. Notably, a higher number of arms, as in 8-arm PEG acrylate, allows more precise control of the mechanical properties.^36,40−42,47−49^ Consequently, we used 8-arm PEG acrylate for all further studies

Furthermore, we made cryogels using different MWs of 8-arm PEGAc (10 and 20 kDa). In our hands, we could only obtain hybrid DN cryogels when using 8-arm PEGAc at 10 kDa. While cryogels could not be formed using 20 kDa 8-arm PEGAc at −12 °C and gels made at −20 °C were mechanically weak (Table S1). Possibly, the lower MW 8-arm PEGAc has faster reaction kinetics and can undergo Michael addition under frozen conditions. However, as the MW of the PEG-acrylate increases, the diffusivity and, hence, the reaction kinetics are hindered under frozen conditions, leading to no or incomplete reaction between the PEG-acrylate and cross-linkers; thus, a cryogel is not obtained.^46^

Last, the concentrations of PEG and alginate polymer networks and the cross-linking agent were optimized. First, two different concentrations of alginate were tested: 1% and 1.5% w/v alginate. When the alginate solution concentration exceeded 1.5%, it became excessively viscous, leading to the trapping of bubbles and nonhomogenous mixing. Thus, only 1% (w/v) alginate for used for cryogel synthesis. In the case of alginate network, CaCl_2_ was initially used as the cross-linker. However, due to the instant reaction, it was difficult to control the gelation of alginate before freezing. Thus, CaCO_3_ and gluconolactone (GDL) were subsequently used as cross-linkers for the alginate network. The use of CaCO_3_ as the cross-linker and GDL as the hydrolyzer allows for slow gelation (15 to 30 min depending on the molar concentration) of the alginate network.^50−53^ The slower gelation kinetics allowed enough time to mix the two networks adequately, freeze them while the gelation did not occurr, and form homogeneous cryogels.

10% and 20% w/v 8-arm PEGAc (10 kDa) were used for the second network. The cryogels prepared with 20% 8-arm PEGAc were opaque, maintained their integrity, and had higher mechanical strength than those prepared with 10% w/v 8-arm PEGAc, as expected. Thus, 8-arm PEGAc was used at a 20% w/v concentration for the synthesis of hybrid DN cryogels. Finally, hybrid DN cryogels could be obtained using any of the three dithiol cross-linkers: DTT, DTBA, and EGBMA. Further studies aimed at characterizing and comparing the properties of hybrid DN cryogels in which the PEG network was cross-linked via one of the three dithiol cross-linkers while the second network was always composed of 1% w/v alginate. Henceforth, we named the hybrid DN cryogels cross-linked with different dithiol cross-linkers as DTT-cross-linked, DTBA-cross-linked, or EGBMA-cross-linked cryogels.

### 3.2 Microstructure of Cryogels

**Figure 2:**
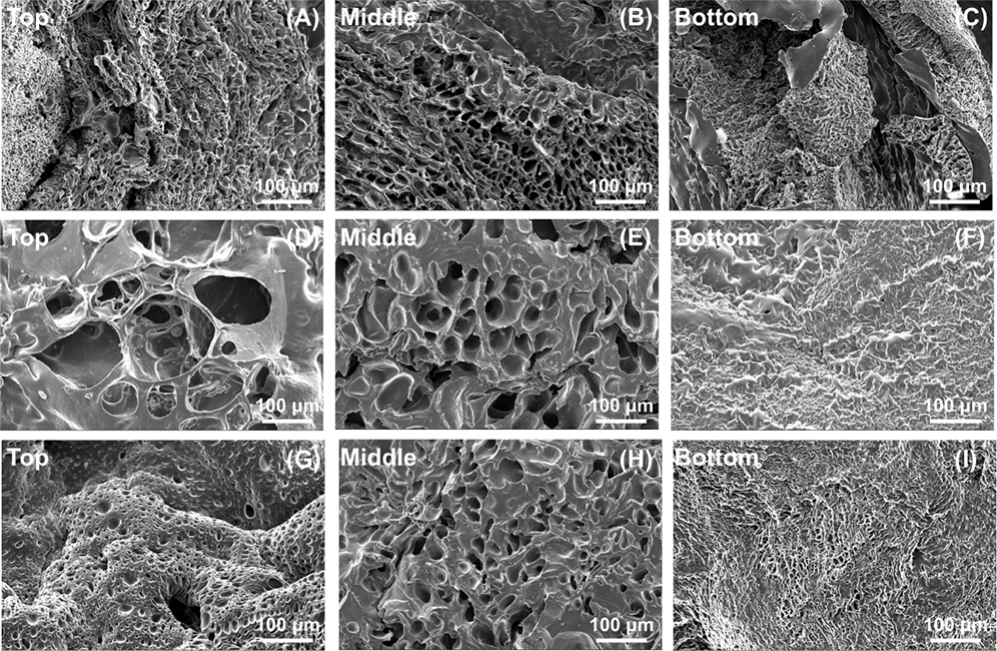
Scanning electron microscopy images of PEG-alginate hybrid DN cryogels. Top, middle, and bottom cross-sections were imaged for each cryogel. (A–C) DTT-cross-linked PEG-alginate hybrid DN cryogels, (D–F) DTBA-cross-linked PEG-alginate hybrid DN cryogels, and (G–I) EGBMA-cross-linked PEG-alginate hybrid DN cryogels.

**Figure 3:**
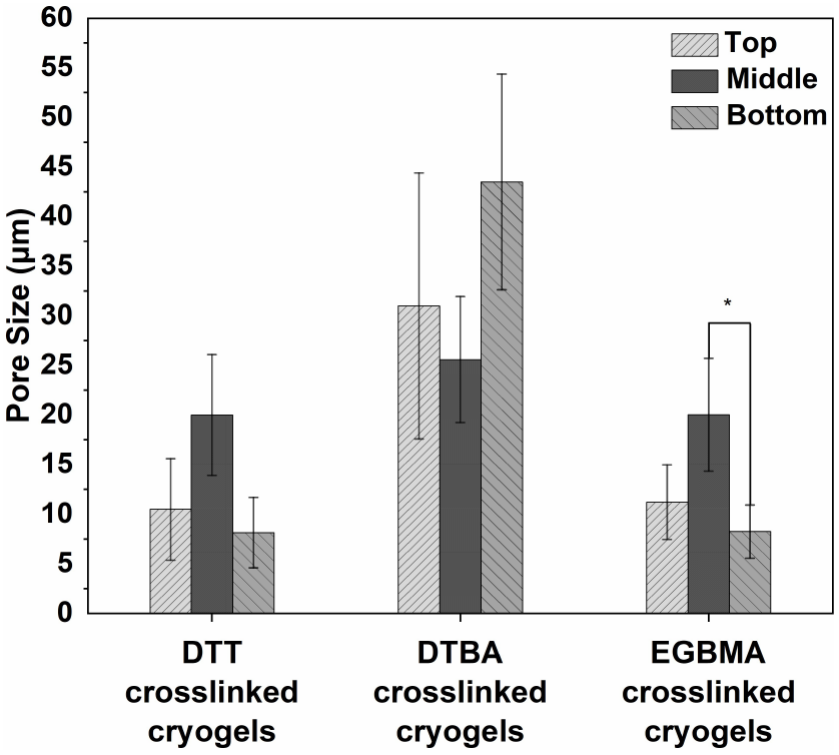
Pore size distributions in the top, middle, and bottom cross-sections of the PEG-alginate hybrid DN cryogels. Pore size distribution among different cryogels was compared using one-way ANOVA, with Tukey’s correction; * indicates *p* < 0.05 (*n* = 3).

SEM images of the PEG-alginate DN hybrid cryogels made using the three different dithiol cross-linkers are shown in Figures 2 and S1. We found that all three hybrid DN cryogels prepared using the different dithiol cross-linkers had a macroporous interconnected structure with a pore size ranging between 4 and 40 μm, although they had distinctly different surface morphologies (Figure 2). Overall, the cryogels prepared using DTBA as the cross-linker seemed to have a higher pore size than the (Figures 2 and 3) the other two cryogels. However, the difference was not statistically significant. Furthermore, the pore size varied slightly in the top, middle, and bottom cross-sections of each cryogel sample. Our results indicate that the pore size in the top cross-section is no different from that in the middle and bottom (Figure 3); only the middle part of cryogels prepared using EGBMA have a higher pore size than the bottom, which may be due to the minor temperature gradient inside the cryogels, whereby the middle portion of the cryogel experiences a slightly higher temperature than the bottom; thus, it may be more conducive to the formation of larger pore structures.^54^ Nonetheless, this pore size range is found to be suitable for chondrocyte culture and supports neocartilage formation.^17,55,56^

### 3.3 Swelling Kinetics of Cryogels

**Figure 4:**
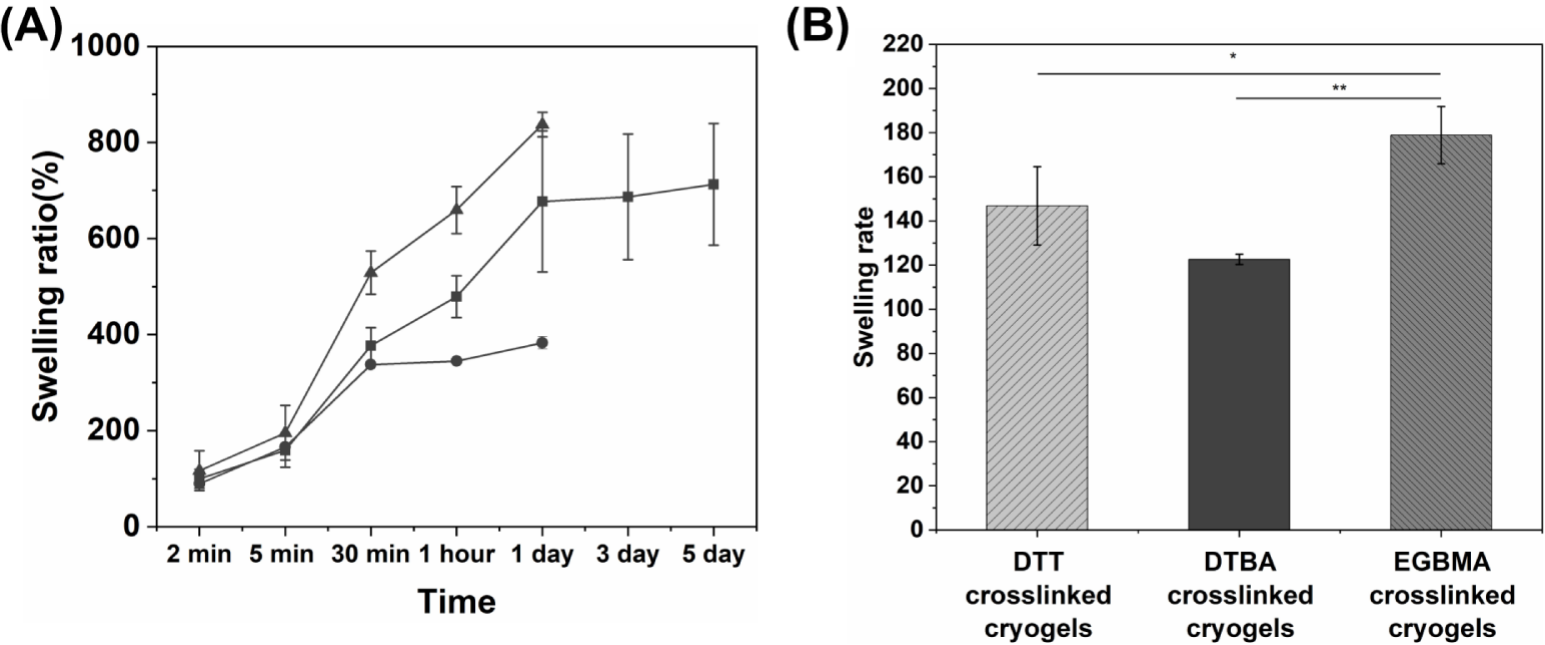
Swelling kinetics of the PEG-alginate hybrid DN cryogels. (A) Swelling ratios of the PEG-alginate hybrid DN cryogels cross-linked with either (box solid) DTT, (circle solid) DTBA, or (triangle up solid) EGBMA dithiol cross-linker. (B) Swelling rates of PEG-alginate hybrid DN cryogels. All statistical analyses were conducted using one-way ANOVA with Tukey’s correction; * indicates *p* < 0.05; ** indicates *p* < 0.01 (*n* ≥ 3 cryogels).

All the three cryogels made using different dithiol cross-linkers swelled rapidly after soaking in PBS for 2 min, and the swelling ratio reached approximately 100% (Figure 4). The DTBA cross-linked PEG-alginate hybrid DN cryogel attained equilibrium within 30 min with a swelling ratio of approximately 350%. However, after 24 h, the swelling ratio of DTBA cross-linked PEG-alginate hybrid DN cryogel was significantly lower than the other two cryogels. The DTT cross-linked PEG-alginate hybrid DN cryogel required more than 24 h to reach equilibrium and achieved a swelling ratio of approximately 670%. The equilibrium time for the EGBMA cross-linked PEG-alginate hybrid DN cryogels could not be determined because the swelling ratio of this cryogel continued to increase even after 24 h. These differences in the swelling behavior of the three cryogels could be due to their reaction efficiency under subzero conditions and their susceptibility for hydrolytic degradation. Previous work by our group has shown that the reaction efficiency is impacted by the presence of different functional groups in the vicinity of the end thiol in dithiol cross-linkers. Moreover, our previous studies showed that DTT has the highest cross-linking efficiency compared to DTBA due to differences in the functional groups near the end thiol (Figure 1D).^41^ This may lead to a higher cross-link density and denser polymer wall formation during cryogelation, resulting in slower swelling kinetics for DTT cross-linked PEG-alginate hybrid DN cryogels. In the case of EGBMA cross-linked cryogels, high susceptibility to hydrolytic degradation leads to faster swelling kinetics, as evident by gel degradation as early as 24 h post incubation (Figure 4). In cryogels, most of the water is absorbed by the capillaries forming the interconnected pore network rather than by the polymer itself; thus, in general, cryogels have a shorter swelling time and reach equilibrium quickly.^57,58^ Rapid equilibration with the surrounding medium is a major advantage of using cryogels as cell scaffolds as this minimizes the equilibration time of the cryogels with the medium and allows efficient nutrient transport.^26,59^

### 3.4 Degradation Kinetics of the Cryogels

**Figure 5:**
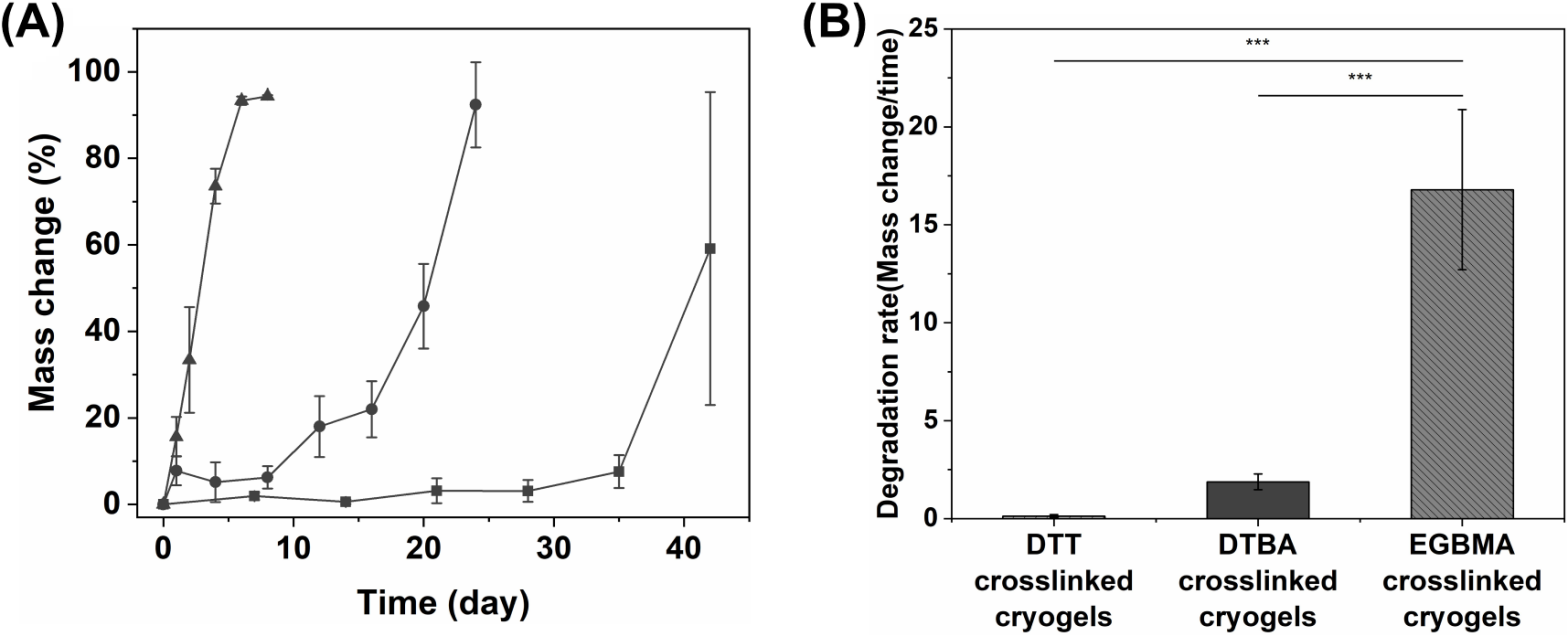
Degradation kinetics of the PEG-alginate hybrid DN cryogels. (A) Degradation kinetics of the PEG-alginate hybrid DN cryogels cross-linked with either (box solid) DTT, (circle solid) DTBA, or (triangle up solid) EGBMA dithiol cross-linker. (B) Degradation rates of the PEG-alginate hybrid DN cryogels. All statistical analyses were conducted using one-way ANOVA with Tukey’s correction; *** indicates *p* < 0.001 (*n* ≥ 3 cryogels).

Among the three types of cryogels, EGBMA cross-linked cryogels exhibited the fastest degradation rate, while DTT cross-linked cryogels took the longest time to degrade (Figure 5). For EGBMA cross-linked cryogels, the mass change occurred at a constant rate every 24 h. Comparatively, DTT and DTBA cross-linked cryogels degraded slowly during the initial period and showed a rapid mass change during the later period close to their degradation times. EGBMA cross-linker has an ester group near the end thiol, which results in extra esters (4 instead of 2) per cross-link during the gel network formation. Moreover, the close proximity of the ester group to the terminal thiol increases the hydrolytic susceptibility to attack by hydronium water ion.^41^ This leads to the rapid degradation of the EGBMA cross-linked PEG network within the hybrid DN cryogel.^60^ DTT has a hydroxyl group (−OH) close to the terminal thiol, which is electron withdrawing in nature. While in DTBA, the group close to the terminal thiol is an electron-donating amino group (−NH2) (Figure 1D). This structural difference in the cross-linkers affects the cross-link density, leading to differences in the rate of hydrolysis reaction at each cross-link. As discussed above, previous work by us has shown that DTT has a high cross-linking efficiency and density, thus, causing DTT-cross-linked cryogels to have a relatively slower degradation rate.^41^ Comparatively, DTBA has a lower cross-linking efficiency and thus lower cross-link density, leading to faster degradation of DTBA-cross-linked cryogels. Thus, we were able to control the rate of degradation of the hybrid DN cryogels using small-molecule dithiol cross-linkers of similar molecular weight but possessing different functional groups near the terminal thiol. PEG itself does not possess reactive functional groups or hydrolysis or enzyme degradation sites. Therefore, we used cross-linkers with varying chemical identities to make PEG hydrogel networks degradable in physiologically relevant environments. Designing scaffolds with similar chemical components but different degradation rates can be beneficial for cell culture as they may allow cells to remodel their environment at desired rates. Previous studies have shown that degradability and degradation rates of scaffolds are critical for chondrocyte growth and remodeling of their microenvironment.^6,16,61,62^ Thus, the macroporous structure of cryogels can provide adhesion and proliferation sites for chondrocytes. Meanwhile, different degradation rates of the cryogel scaffolds can be matched to promote the formation of neocartilage.^63^

### 3.5 Compressive Mechanical Properties of Hybrid DN Cryogels

Compression tests were performed to evaluate the effect of different cross-linkers on the compressive mechanical properties of the cryogels. These results are summarized in Table 2. We analyzed the linear region of the slope of the stress–strain curve to obtain the compressive modulus of hybrid DN cryogels cross-linked with either DTT, DTBA, or EGBMA. Our results indicate that the hybrid DN cryogels cross-linked with EGBMA had the highest compressive modulus. Both DTBA and EGBMA cross-linked cryogels had a higher compressive modulus than the DTT cross-linked cryogels but exhibited 34–40% deformation, indicating their brittle nature. While the DTT cross-linked cryogels had the lowest modulus and highest toughness. These differences in the compressive mechanical properties can be attributed to the differences in the cross-linking density, which is dependent on the chemical properties of the dithiol cross-linker used for cryogel formation. Moreover, the hybrid double-network cryogels synthesized in this study have higher mechanical strength than PEG or alginate single-network cryogels reported in the literature.^31,34,36,64^ Since cartilage is a load-bearing tissue, the mechanical properties of a scaffold for cartilage tissue engineering are considered extremely important.^6,65^

**Table 2:** Compression Mechanical Properties of the PEG-Alginate Hybrid DN Cryogels

PEG-alginate cryogel cross-linked with	toughness (kJ/mm^3^)	stress at break (kPa)	strain at break (%)	compressive modulus (kPa)
DTT	15.9 ± 4.99	84.7 ± 23.5	59.2 ± 4.6	54.80 ± 10.2
DTBA	7.1 ±1.02^a^^,^^b^	47.2 ± 4.5^a^^,^^b^	39.4 ± 3.9^a^	188.70 ± 21.2^c^^,^^d^
EGBMA	10.4 ± 2.88	73.7 ± 17.5	34.9 ± 2.1^a^	388.54 ± 51.9^e^

a Indicates *p* < 0.05.

b Indicates *p* < 0.05 or.

c *p* < 0.01 or.

d *p* < 0.001 when compared to EGBMA-cross-linked cryogels.

e *p* < 0.0001 when compared to DTT-cross-linked cryogels.

### 3.6 Rheological Properties of the Hybrid DN Cryogels

**Figure 6:**
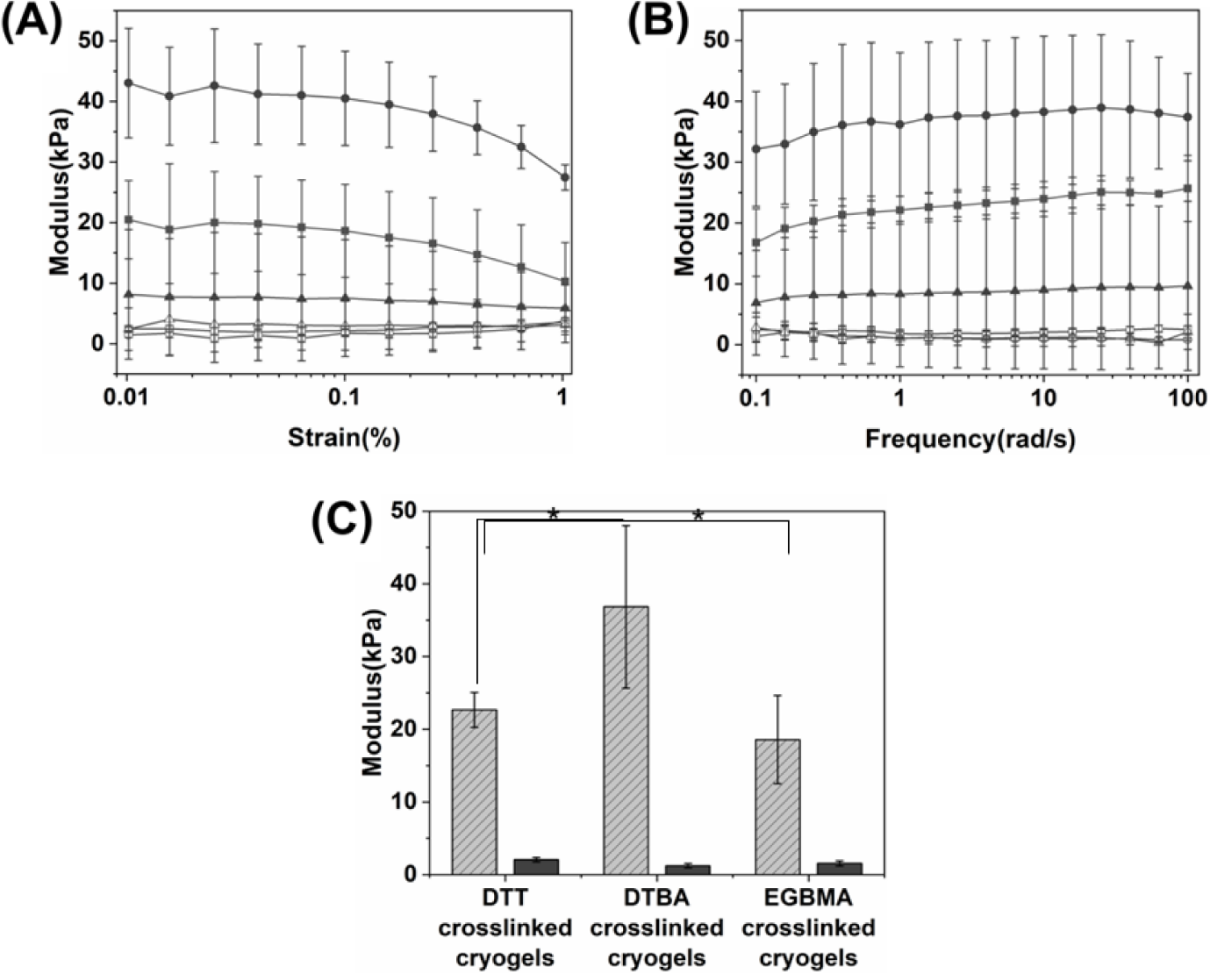
Rheological analysis of the PEG-alginate hybrid DN cryogels cross-linked by different dithiol cross-linkers: (A) Representative storage modulus (*G*′) (filled markers) and loss modulus (*G*′’) (open markers) of the (box solid) DTT-, (circle solid) DTBA-, or (triangle up solid) EGBMA-cross-linked PEG-alginate hybrid DN cryogels as measured by strain amplitude test. (B) Representative storage modulus (*G*′) (filled markers) and loss modulus (*G*′’) (open markers) of the (box solid) DTT-, (circle solid) DTBA-, or (triangle up solid) EGBMA cross-linked PEG-alginate hybrid DN cryogels as measured during oscillatory frequency test. (C) The average storage modulus (*G*′) and loss modulus (*G*′’) of the DTT, DTBA, or EGBMA cross-linked PEG-alginate hybrid DN cryogels measured at 0.1% strain in a frequency range of 1–100 rad/s. * indicates *p* < 0.05 (*n* ≥ 3).

First, to determine the linear viscoelastic region of cryogels, strain amplitude tests were conducted in the strain range of 0.01–1% (Figure 6). Within the strain range of 0.01–0.1%, the storage modulus of each cryogel remained constant with increasing strain. As the strain increased to 1%, the storage moduli started to decline. Interestingly, before the intersection of the storage modulus and loss modulus, with increasing strain, the elastic portion mainly decreased, and the viscous portion increased very slowly. This may be due to a delay in the breakdown of the structure during crack propagation as the water retained within the pore walls exudes from the cryogels.^66^ Thus, a fixed strain of 0.1% was chosen for the frequency amplitude test.

At a fixed strain of 0.1%, all cryogels had a similar loss modulus of ∼1.5 kPa, which was atleast 10 times lower than the storage modulus, indicating stable gel formation and elastic nature of the cryogels. The storage modulus of the DTBA cross-linked cryogels was found to be significantly higher than that of the EGBMAor DTT cross-linked cryogels. The differences in the storage moduli of the three hybrid DN cryogels may be attributed to minor differences in their chemical structures. Multiple factors can affect storage modulus, like cross-link density, water uptake ability, and temperature.^67^ The hybrid DN cryogels formed with different cross-linkers can have a difference in gel fractions, which is related to their gelation rate or reaction efficiency. Besides, the three cryogels have different water absorption capabilities, resulting in differences in the final storage modulus.^67^ In the case of DTBA cross-linked cryogels, there may be additional noncovalent interactions between the positively charged pendant amine groups in DTBA and the negatively charged carboxyl groups in alginate. Such interactions may be absent in the EGBMAand DTT cross-linked cryogels, leading to a lower storage modulus.

Viscoelasticity is an important criterion for scaffolds to be used for cartilage tissue engineering. Recent research indicates that the viscoelasticity of scaffolds plays a dynamic role in regulating cell differentiation during various stages of chondrogenesis.^68^ Thus, the PEG-alginate DN cryogels with similar storage modulus and pore size but different degradation rates can be used to study the effects of these parameters on cell fate and cell-matrix interactions.

### 3.7 Release Kinetics of TGF-β1 and IGF-1 from Hybrid DN Cryogels

**Figure 7:**
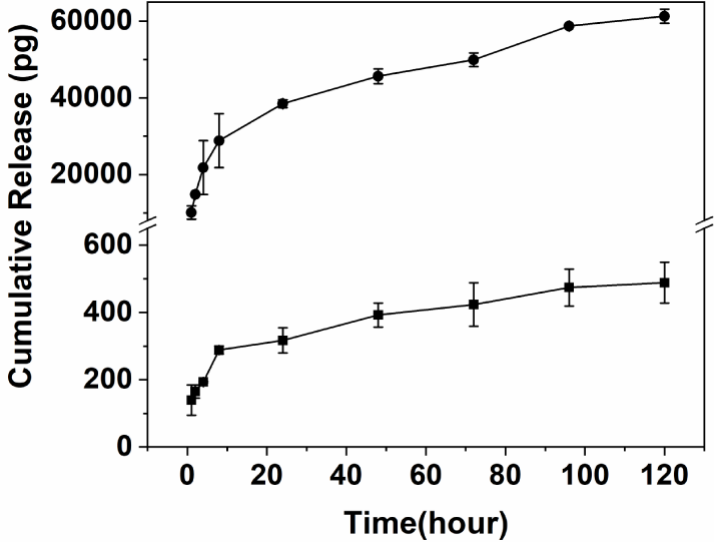
Cumulative release of the growth factors: (box solid) TGF-β1 and (circle solid) IGF-1 from DTBA cross-linked PEG-alginate hybrid DN cryogel. The amount of growth factors released daily was measured by ELISA for 7 days. Cumulative release was calculated by adding daily release values. Results are presented as the mean value ± standard deviation (*n* ≥ 3).

To assess the suitability of cryogels as a potential scaffold for cartilage tissue engineering, we assessed their ability for the sustained release of growth factors critical for cartilage regeneration. TGF-β1 and IGF-1 were loaded onto DTBA cross-linked cryogels, and their release was studied over a 7-day period (Figure 7). The final loading for TGF-β1 and IGF-1 in cryogels was 93 ± 2.2 and 93.9 ± 3.3 ng, respectively. This indicates a high loading efficiency of >90% for the two growth factors. Both IGF-1 and TGF-β1 could be released sustainably from the cryogels for 7 days. Compared to IGF-1, TGF-β1 was released at a slower rate, and only 0.2% (200 pg) was released in the first 8 h, reaching a peak on day 5, after which the release rate reached a plateau. In the case of IGF-1, 13% was released in the first hour, and then, the release continued to increase steadily until day 7, when all the loaded IGF-1 was released from the cryogels. The difference in release kinetics of the two growth factors can be attributed to the differences in their molecular weight and affinity for the cryogel matrix. TGF-β1 is a 25-kDa protein and is ∼3 times the MW of IGF-1 (7.6 kDa).^69,70^ Furthermore, charge-based interactions between alginate and growth factors^71^ can possibly slow down their diffusion from the macroporous cryogels. Our results indicate that the PEG-alginate DN cryogels have a higher retention capacity for TGF-β1, indicating a stronger interaction between the cryogel and TGF-β1. Nonetheless, the sustained release of these critical growth factors can potentially accelerate chondrogenesis and help in the formation of neocartilage. Many studies suggest that scaffolds with immobilized or encapsulated growth factors, especially TGF-β1, have high potential for chondrogenic differentiation of stem cells. TGF-β1 has been deemed crucial for the onset and maintenance of chondrogenesis in stem cells.^72^ IGF-1, on the contrary, promotes chondrocyte proliferation and cartilage matrix synthesis.^73^ Thus, scaffolds that can control the release of such critical growth factors have a high appeal for articular cartilage repair. Our results suggest that PEG-alginate DN cryogels prepared in this study have a high potential for sustained delivery of growth factors that are crucial for cartilage regeneration.

### 3.8 Cell Culture in the PEG-Alginate Hybrid DN Cryogels

**Figure 8:**
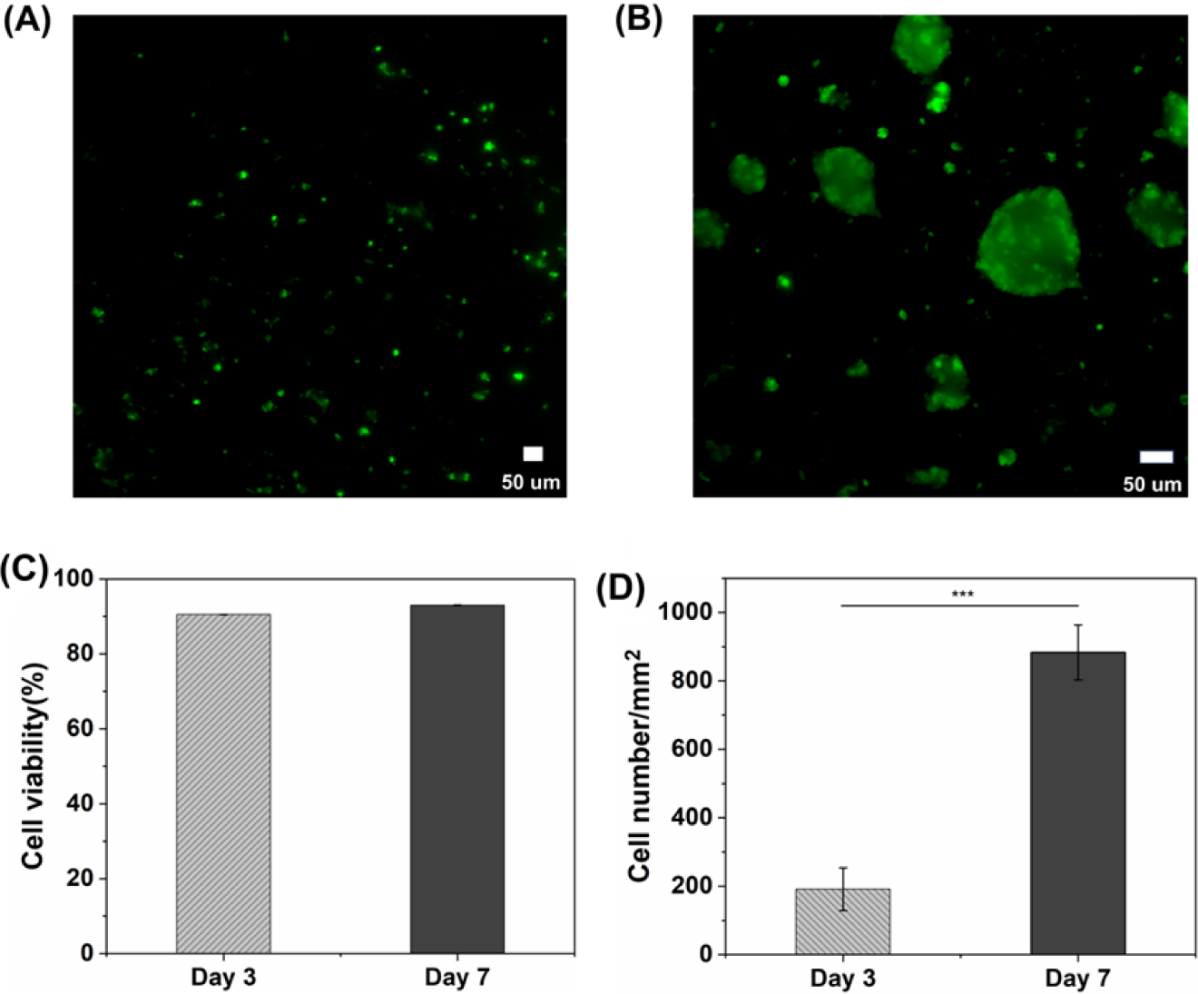
Culture of D1 cells in cryogels. Live/dead staining of D1 cells seeded on DTBA cross-linked PEG-alginate hybrid DN cryogels on (A) day 3 and (B) day 7. (C) Percent viability of D1 cells seeded on DTBA cross-linked hybrid DN cryogels on day 3 and day 7. (D) The average cell density of D1 cells infiltrating the top, middle, and bottom cross-sections of DTBA hybrid DN cryogels on day 3 and day 7. Results are presented as the mean value ± standard deviation (*n* ≥ 3). Statistical significance between the two groups was calculated using a *t* test. *** indicates *p* < 0. 001.

DTBA cross-linked cryogels were coated with collagen before cell seeding to provide sites for cell attachment. D1 cells were chosen as the model cells because prior research demonstrated their similarity to human mesenchymal stem cells (MSCs) in terms of their cell-matrix responses.^74^ The cells maintained approximately 90% viability on day 3 after cell seeding and continued to maintain this viability until day 7 (Figure 8 A–C). Furthermore, the cells were able to infiltrate the cryogel scaffold efficiently, as indicated by the average cell density in the top, middle, and bottom sections of the cryogel scaffolds (Figure 8D). Cell infiltration in the cryogel scaffolds was obtained by quantifying the number of nuclei stained with DAPI per square millimeter. Representative images (Figure S2) are provided in Supporting Information. These results indicate that the macroporous nature of the scaffolds allows for efficient and uniform cell seeding, thereby, achieving high cell density.

### 3.9 Chondrogenic Differentiation of Mouse MSCs in Hybrid DN Cryogels

**Figure 9:**
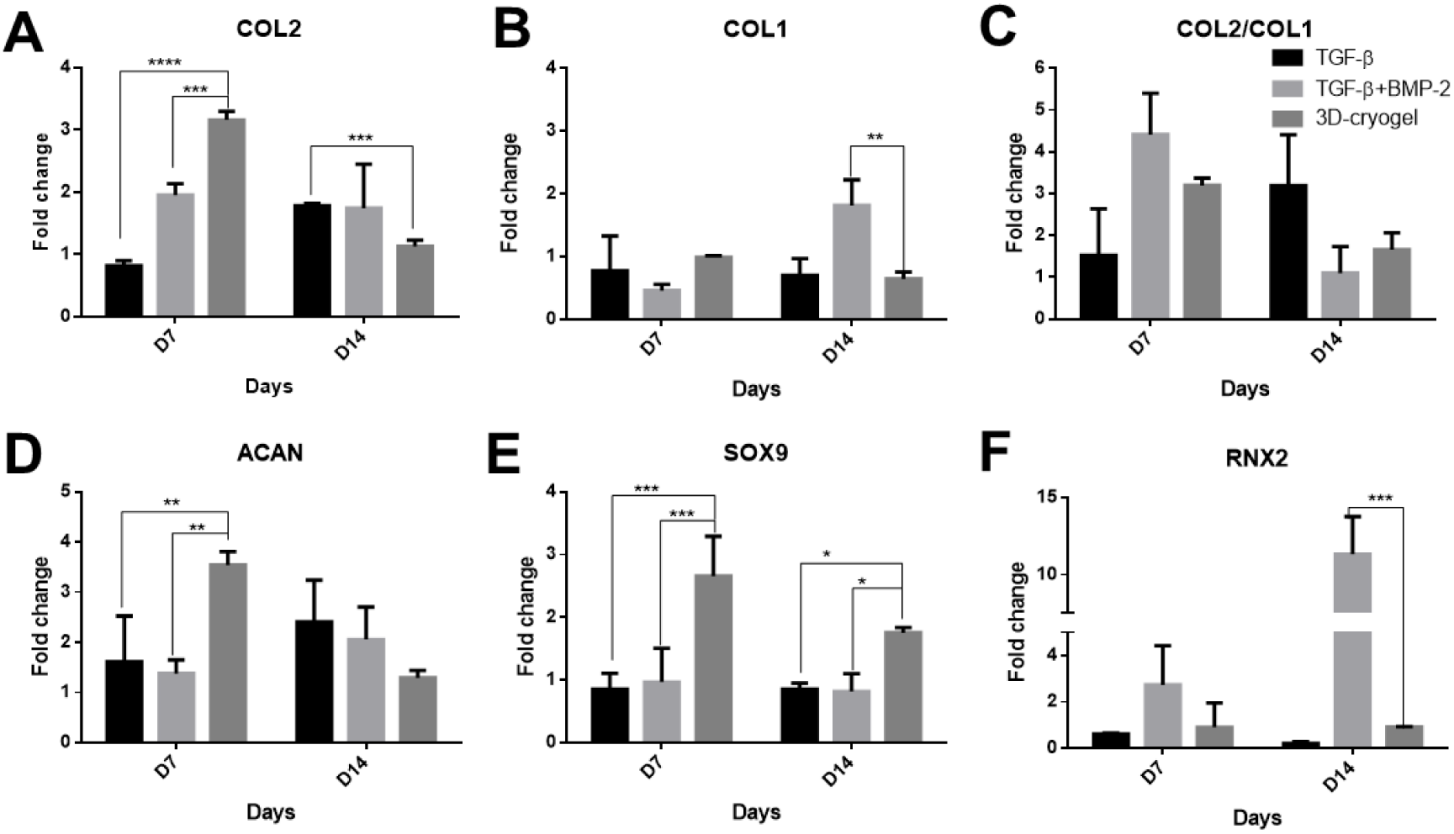
Changes in the gene expression of (A, C, D, and E) pro-chondrogenic and (B and F) hypertrophic markers in D1 MSCs cultured in PEG-alginate hybrid DN cryogels. 3D cryogel indicates cells cultured in DMEM-F12 media without the addition of any exogenous growth factors. TGF-β1 and TGF-β1+ BMP-2 indicate cells cultured in either TGF-β1 or TGF-β1+ BMP-2–supplemented chondrogenic media, respectively. Two-way ANOVA (treatment and time as parameters) with Sidak’s multiple comparison test was used to determine statistically significant difference between the three conditions. 3D cryogel was used as a control to compare different conditions at a given time. All data are represented as mean ± standard deviation (*n* = 3). * indicates *p* < 0.05; ** *p* < 0.005.

The ability of the PEG-alginate hybrid DN cryogels to support the differentiation of D1MSCs toward chondrogenic lineage was evaluated by using gene expression analysis. Gene expression analysis for prochondrogenic markers and hypertrophy markers was conducted in the absence and presence of chondrogenesis-promoting growth factors TGF-β1 and TGF-β1 + BMP-2. D1 cells cultured in hybrid DN cryogels (3D cryogel) without any growth factors showed signs of early chondrogenesis by day 7 (Figure 9 A,D, and E), as indicated by the upregulation of the prochondrogenic markers^75−77^ COL2 (a major component of cartilage ECM), SOX-9 (a transcription factor that regulates chondrogenic differentiation), and ACAN (a cartilage-specific ECM component). While the hypertrophy markers^75−77^ COL1 (indicates ECM secretion by other cell types like fibroblast or undifferentiated cells) and RUNX 2 (a marker for hypertrophy and osteogenic differentiation)^44,78^ were downregulated. Additionally, the ratio of COL2/COL1 was high in 3D cryogel scaffolds without any growth factors (Figure 9C). As MSCs differentiate into chondrocytes, the expression of COL2 increases while that of COL1 decreases. Thus, a high COL2/COL1 ratio indicates chondrogenic differentiation.

In cultures supplemented with TGF-β1 and TGF-β1 + BMP-2, the chondrogenic gene markers were upregulated by day 7, albeit to a lesser extent compared with the 3D cryogel only condition. Furthermore, by day 14, the expression of chondrogenic-specific markers increased in TGF-β1–supplemented cultures compared to day 7, whereas it decreased in 3D cryogels without any growth factors (Figure 9A,D, and E). These results indicate that while embedding cells in a 3D cryogel scaffold alone, in the absence of any growth factors, is sufficient to initiate chondrogenic differentiation, supplementation of TGF-β1 is necessary for sustained differentiation into the chondrogenic lineage.^76^ Cells cultured in TGF-β1 + BMP-2–supplemented chondrogenic media also induced early chondrogenesis by day 7, as indicated by the upregulation of COL2 and a high COL2/COL1 ratio (Figure 9A**–**C). However, by day 14, the expression of the hypertrophy markers COL1 and RUNX 2 increased significantly (Figure 9 B,C, and F). While the addition of BMP-2 synergizes with TGF-β1 to enhance chondrogenic differentiation, it also induces osteogenic differentiation and promotes hypertrophy.^77,79^ Our results here validate these findings from the literature, indicating that while BMP-2 synergizes with TGF-β1 to induce early chondrogenesis, its sustained presence leads to an increase in hypertrophy. Nevertheless, the PEG-alginate DN cryogels synthesized in this study support the differentiation of MSCs into chondrocytes in both the absence and presence of exogenously added growth factors.

**Figure 10:**
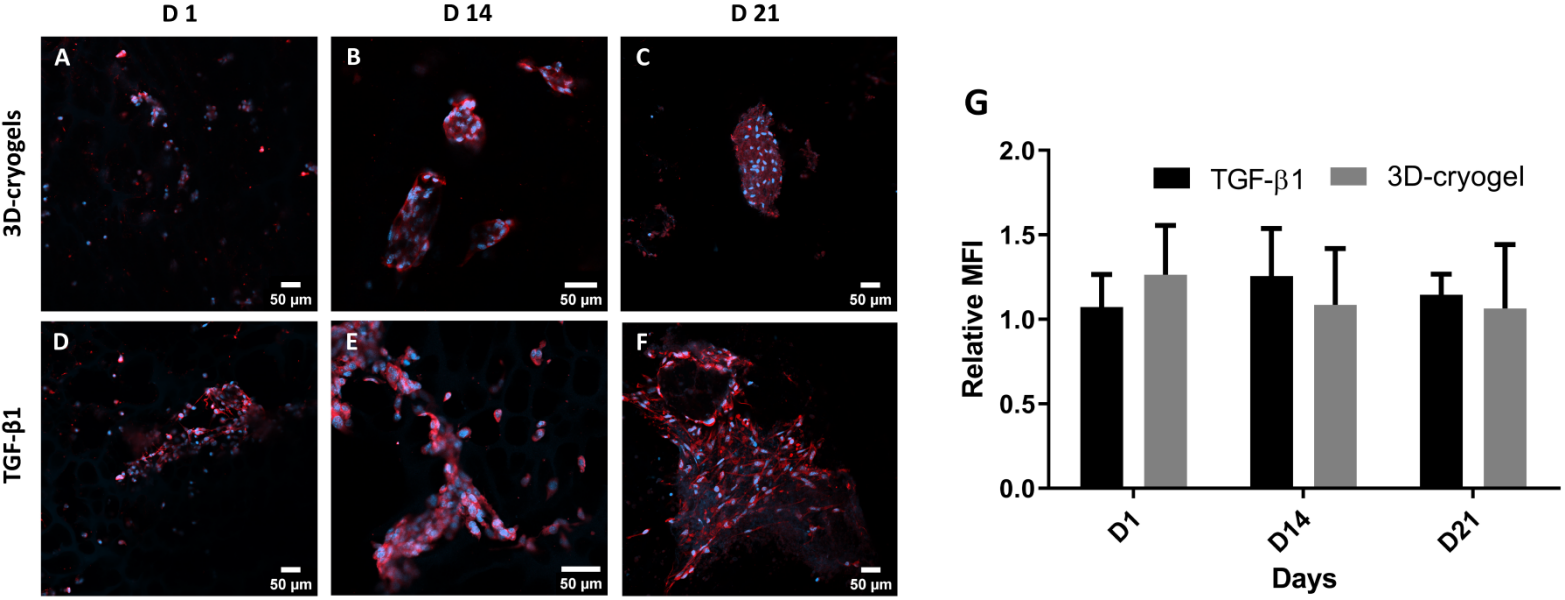
Immunostaining for collagen 2 in D1 MSCs cultured in the PEG-alginate hybrid DN cryogels. Representative images showing collagen 2 immunostaining on days 1, 14, and 21 for cells cultured in 3D cryogel scaffolds (A–C) without TGF-β1 supplementation and (D–F) with TGF-β1 supplementation. (G) Relative mean fluorescent intensity (MFI) for collagen 2 at each time point and under different culture conditions. The relative MFI was determined by obtaining a ratio of the fluorescent intensity for collagen 2 to DAPI. Red indicates collagen 2 staining, and blue indicates staining for nucleus (DAPI).

Furthermore, we compared collagen 2 expression on days 1, 14, and 21 for cells cultured in hybrid DN cryogels with and without TGF-β1 supplementation. We observed the expression of collagen 2 in both TGF-β1 and 3D cryogels at all time points. No statistically significant differences in collagen 2 immunostaining were observed between samples with or without TGF-β1 supplementation at various time points (Figure 10). These results further corroborate our findings that D1 cells cultured in 3D hybrid DN cryogels undergo chondrogenic differentiation with or without TGF-β1 growth factor supplementation. Collectively, these preliminary results from gene expression studies and collagen 2 immunostaining indicate that the PEG-alginate hybrid DN cryogels support MSC culture and chondrogenic differentiation.

## 4 Conclusion

Macroporous PEG-alginate hybrid DN cryogels could be successfully synthesized with similar chemical composition but different degradation rates by judicious choice of dithiol cross-linkers for the PEG network. We were the first to synthesize the PEG-alginate hybrid DN network cryogels using radical-free cross-linking chemistry for both PEG and alginate networks. The ability to control the properties of the macroporous network by varying the cross-linker type provides a facile and novel way to manipulate the structural properties and degradation behavior of such materials. We also found that the mechanical and swelling properties of the cryogels were strongly dependent on the type of dithiol cross-linker used for the PEG network formation.

The hybrid DN cryogels, characterized by interconnected macroporous structures, tunable structural and mechanical properties, rapid swelling kinetics supporting enhanced mass transport of nutrients, and the ability to retain growth factors and control their release, along with their capability to support MSC growth and chondrogenic differentiation, render them excellent scaffolds for cartilage tissue engineering. Thus, this study provides a novel method to generate macroporous hybrid DN cryogels with customizable degradation rates and a potential biocompatible scaffold for cartilage tissue engineering.

## Supporting Information Available

Preliminary screening of synthesis parameters for PEG-alginate hybrid DN cryogels (Table S1); scanning electron microscopy images of PEG-alginate hybrid DN cryogels (Figure S1); representative images showing nuclear staining via DAPI in D1 cells seeded in DTBA cross-linked PEG-alginate hybrid DN cryogels (Figure S2) (PDF)
