# Supplementary material for: Macroporous PEG-Alginate Hybrid Double-Network Cryogels with Tunable Degradation Rates Prepared via Radical-Free Cross-Linking for Cartilage Tissue Engineering
Source: ACS Appl Bio Mater. 2024 Aug 13;7(9):5925–38. doi: 10.1021/acsabm.4c00091 (PMC11409214; doi:10.1021/acsabm.4c00091)
Supplement: Supplementary file 1 — mt4c00091_si_001.pdf [file mt4c00091_si_001.pdf]

Supporting Information

Macroporous PEG-Alginate Hybrid Double-Network Cryogels with Tunable Degradation Rates Prepared via Radical Free Crosslinking for Cartilage Tissue Engineering

*Kaixiang Zhang1,2&, Zining Yang1,2& #, Michael Patrick Seitz1,2, and Era Jain, PhD1,2**

1 Department of Biomedical and Chemical engineering,2 Bioinspired Syracuse: Institute for Material and Living System, Syracuse University, Syracuse, NY

kzhang57@syr.edu, zyang24@syr.edu, mseitz@syr.edu, erjain@syr.edu

***Corresponding Author**

Era Jain (Ph.D.)
Biomedical and Chemical Engineering
Bioinspired Syracuse: Institute for Material and Living System
Syracuse University
Syracuse, NY, USA, 13244
Tel: 315.443.4050
Email: erjain@syr.edu

& denotes equal authorship

# Present Address: Department of Bioengineering, University of Texas, Arlington, TX, USA

Table S1: Preliminary screening of synthesis parameters for PEG-alginate hybrid DN cryogels.

**Cryogel Components**		**Synthesis Temperature**	**Cryogel properties**	
**Alginate concentration and crosslinker**	**Multi-arm PEG acrylate**		**Porosity**	**Qualitative mechanical properties**
1% w/v Alginate + $CaCl_2$	20 % w/v 4-arm PEG acrylate (10 kDa) + DTT	-12 °C	moderate porosity	Can form gels, poor mechanical integrity
1.5% w/v Alginate + $CaCl_2$	10 % w/v 4-arm PEG acrylate (10 kDa) + DTT	-12 °C	no pores	Can form gels, poor mechanical integrity
1% w/v Alginate + CaCO3+ GDL	10 % w/v 4-arm PEG acrylate (10 kDa) + DTT	-12 °C	moderate porosity	Can form gels, poor mechanical integrity
1% w/v Alginate + CaCO3+ GDL	10 % w/v 8-arm PEG acrylate (10 kDa) + DTT	-12 °C	moderate porosity	Can form gels, poor mechanical integrity
1% w/v Alginate + CaCO3+ GDL	20 % w/v 8-arm PEG acrylate (20 kDa) + DTT	-12 °C	no pores	Mostly liquid and cannot form gels
1% w/v Alginate + CaCO3+ GDL	20 % w/v 8-arm PEG acrylate (20 kDa) + DTT	-20 °C	moderate porosity	Can form gels, poor mechanical integrity
1% w/v Alginate + CaCO3+ GDL	20 % w/v 8-arm PEG acrylate (10 kDa) + DTT	-20 °C	interconnected macroporous	Yellow, elastic, good mechanical integrity
1% w/v Alginate + GDL	20 % w/v 4-arm PEG acrylate (10 kDa) + DTT	-20 °C	moderate porosity	White, elastic, good mechanical integrity

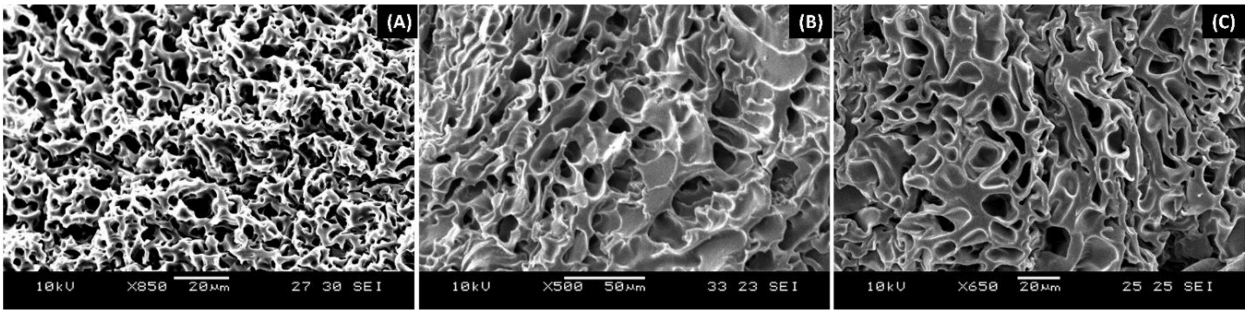

Figure S1: Scanning electron microscopy images of PEG-alginate hybrid DN cryogels at 500X magnification. **(A)** DTT crosslinked PEG-alginate hybrid DN cryogels, **(B)** DTBA crosslinked PEG-alginate hybrid DN cryogels, and **(C)** EGBMA crosslinked PEG-alginate hybrid DN cryogels.

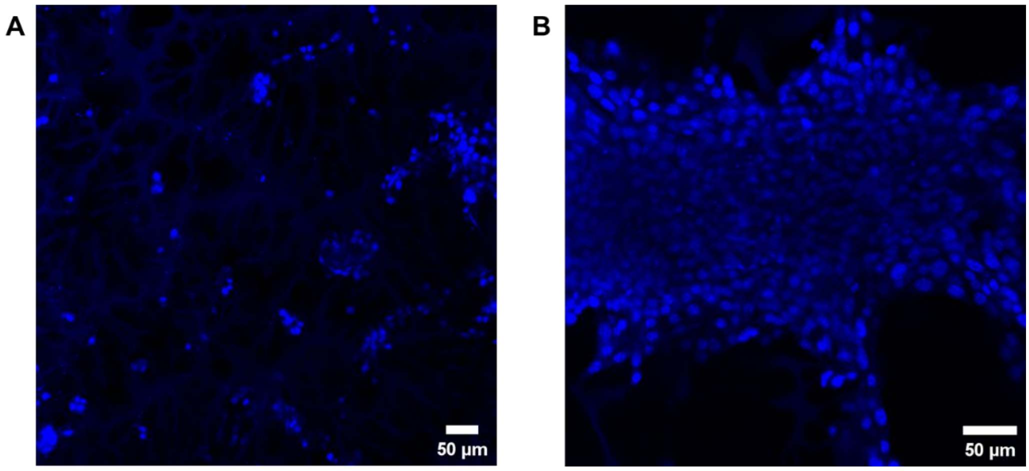

Figure S2: Representative images showing nuclear staining via DAPI in D1 cells seeded in DTBA crosslinked PEG-alginate hybrid DN cryogels. Images of cells on **(A)** day 3 **(B)** day 7
